# Fully gapped pairing state in spin-triplet superconductor UTe_2_

**DOI:** 10.1126/sciadv.adk3772

**Published:** 2024-02-07

**Authors:** Shota Suetsugu, Masaki Shimomura, Masashi Kamimura, Tomoya Asaba, Hiroto Asaeda, Yuki Kosuge, Yuki Sekino, Shun Ikemori, Yuichi Kasahara, Yuhki Kohsaka, Minhyea Lee, Youichi Yanase, Hironori Sakai, Petr Opletal, Yoshifumi Tokiwa, Yoshinori Haga, Yuji Matsuda

**Affiliations:** ^1^ Department of Physics, Kyoto University, Kyoto 606-8502, Japan.; ^2^ Department of Physics, University of Colorado Boulder, Boulder, CO 80309, USA.; ^3^ Advanced Science Research Center, Japan Atomic Energy Agency, Tokai, Ibaraki 319-1195, Japan.

## Abstract

The recently discovered superconductor UTe_2_ is a promising candidate for spin-triplet superconductors, but the symmetry of the superconducting order parameter remains highly controversial. Here, we determine the superconducting gap structure by the thermal conductivity of ultraclean UTe_2_ single crystals. We find that the *a*-axis thermal conductivity divided by temperature κ/*T* in zero-temperature limit is vanishingly small for both magnetic field ***H***‖*a* and ***H***‖*c* axes up to *H*/*H*_*c*2_ ∼ 0.2, demonstrating the absence of nodes around the *a* axis contrary to the previous belief. The present results, combined with the reduction of nuclear magnetic resonance Knight shift, indicate that the superconducting order parameter belongs to the isotropic *A_u_* representation with a fully gapped pairing state, analogous to the B phase of superfluid ^3^He. These findings reveal that UTe_2_ is likely to be a long-sought three-dimensional strong topological superconductor, hosting helical Majorana surface states on any crystal plane.

## Teaser

The thermal transport experiments on ultraclean UTe2 crystals reveal a full-gap spin-triplet superconducting state.

## INTRODUCTION

Spin-triplet pairing states have aroused tremendous interest because of the emergence of Majorana quasiparticles (*1*) and their potential application to fault-tolerant quantum information processing (*2*, *3*). The most famous example is the superfluid ^3^He (*4*), and the quest for its superconducting analog has been a long-standing issue in condensed matter physics. The promising candidate is the recently discovered heavy fermion superconductor UTe_2_ (*5*). UTe_2_ exhibits highly unusual superconducting properties, including extremely large upper critical fields well exceeding the Pauli limit (*5*, *6*), coexistence of superconductivity and ferromagnetic order in high magnetic field (*7*, *8*), reentrant superconductivity that resembles to ferromagnetic superconductors URhGe and UCoGe (*7*, *9*), and peculiar behavior of nuclear magnetic resonance (NMR) Knight shift and absence of coherence peak in the superconducting state (*5*, *10*–*12*). All of these notable properties are indicative of the spin-triplet pairing state. Moreover, this unconventional superconducting state occurs in the paramagnetic state at ambient pressure. This is in contrast to the other ferromagnetic superconductors; at ambient pressure, UGe_2_ shows a ferromagnetic order (*13*), and, in URhGe and UCoGe, superconductivity coexists with ferromagnetic order (*14*, *15*). Therefore, UTe_2_ is a long-sought material that allows us to examine superconducting properties in more detail using various probes.

**Fig. 1.**
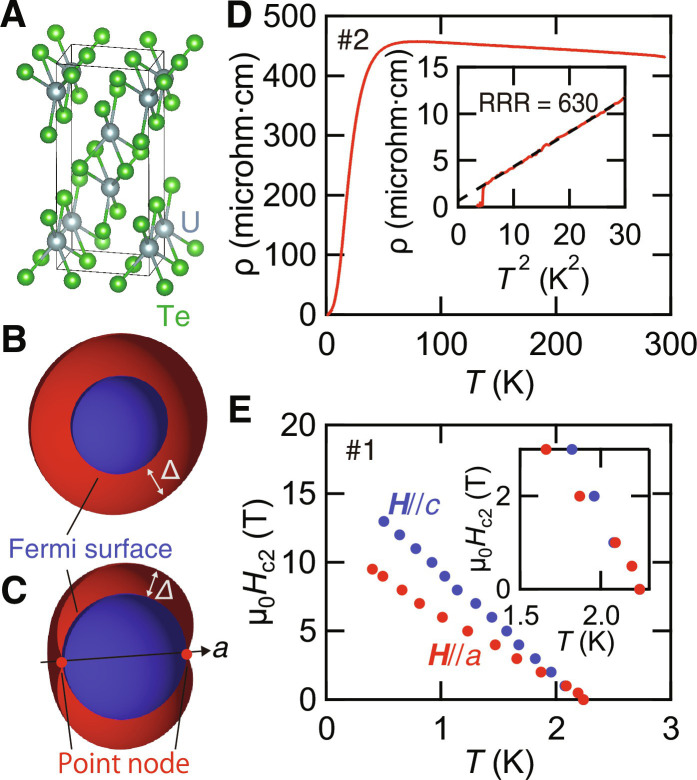
Crystal structure, resistivity, and phase diagram of UTe_2_. (**A**) Crystal structure of UTe_2_. The gray and green spheres represent U and Te atoms, respectively. (**B** and **C**) Structure of the superconducting gap Δ for *A_u_* (B) and *B*_3*u*_ (C) symmetries for spherical Fermi surface (blue sphere). The *B*_3*u*_ state has point nodes (red points) along the *a* axis. (**D**) Temperature dependence of resistivity ρ for sample #2. The inset shows ρ as a function of *T*^2^ at low temperatures. The residual resistivity ρ_0_ is obtained by a fit to ρ(*T*) with ρ_0_ + *AT*^2^ (dashed line). (**E**) *H-T* phase diagram determined by resistivity measurements of sample #1. The linear extrapolations to *T* = 0 yield the upper critical fields μ_0_*H*_*c*2_ of ∼12 T for ***H***‖*a* and ∼17 T for ***H***‖*c*. The inset shows an enlarged view near *T_c_*.

Of particular interest is the superconducting gap structure of UTe_2_, which is of primary importance for understanding the peculiar superconducting state associated with the spin-triplet pairing state. Despite the intensive research efforts, however, the superconducting order parameter has been highly controversial, and its determination is still challenging. At an early stage, a chiral superconducting state with a multicomponent order parameter has been suggested by the double peak of the specific heat and polar Kerr effect (*16*), which was supported by scanning tunneling spectroscopy experiments (*17*). However, as the sample quality improves, a single peak is observed in the specific heat (*18*–*20*) and does not split into two peaks even under uniaxial strain (*21*). Moreover, recent Kerr effect experiments on clean crystals report no evidence of broken time-reversal symmetry (*22*). Considering the orthorhombic crystal structure of UTe_2_ (Fig. 1A), although the chiral state may appear at the surface, chiral superconductivity is unlikely to be realized in the bulk.

Given no solid evidence for the bulk chiral superconductivity in UTe_2_, there are four possible gap symmetries for spin-triplet superconductivity, {*A_u_*, *B*_1*u*_, *B*_2*u*_, *B*_3*u*_} (*23*). The *A_u_* symmetry is fully gapped (Fig. 1B), corresponding to the B phase of superfluid ^3^He. On the other hand, *B*_1*u*_, *B*_2*u*_, and *B*_3*u*_ symmetries have point nodes at isolated points on the Fermi surface along the *c*, *b*, and *a* axes, respectively (Fig. 1C). Several measurements, such as thermal conductivity (*24*), penetration depth (*25*, *26*), and specific heat (*27*), have reported the presence of the low-energy quasiparticle excitations, suggesting the presence of point nodes rather than line nodes. NMR measurements show that, below the superconducting transition temperature *T_c_*, the Knight shift along the *b* and *c* axes decreases (*11*, *12*). This excludes the *B*_1*u*_ and *B*_2*u*_ symmetries. On the basis of these results, it has been widely discussed that the *B*_3*u*_ symmetry with point nodes along the *a* axis accounts for the superconducting gap of UTe_2_. The point node(s) around the *a* axis has also been suggested by thermal conductivity (*24*).

However, it should be stressed that, because the low-energy quasiparticle excitations in spin-triplet superconductors are extremely sensitive to impurities (*28*), the determination of the gap symmetry in the previous measurements may be hindered by impurity. Moreover, as mentioned previously, the gap structure near the surface may be distinct from the bulk. Therefore, it is premature to conclude the presence of point nodes along the *a* axis. Determining whether the gap symmetry is *A_u_* or *B*_3*u*_ is of crucial importance because the topological properties of these two symmetries are fundamentally different (*29*). Thus, the situation calls for reexamining the gap structure using a bulk probe on crystals with an extremely low impurity concentration. Here, we determined the superconducting gap structure of ultraclean UTe_2_ crystals (*30*, *31*) by thermal conductivity κ, which is a bulk and directional probe of the superconducting gap structure (*32*). In contrary to the previous reports, the present results demonstrate the absence of any type of nodes at and around the *a* axis. This indicates that the superconducting order parameter of UTe_2_ belongs to the isotropic *A_u_* representation with a fully gapped pairing state, analogous to the B phase of superfluid ^3^He.

## RESULTS

The superconducting transition temperature *T_c_* of both crystals #1 and #2 is 2.1 K, which is higher than the previously reported value of typically ∼2.0 K (*20*). Figure 1D displays the temperature (*T*) dependence of the resistivity ρ(*T*) along the *a* axis for sample #2. The normal state resistivity is well fitted by ρ(*T*) = ρ_0_ + *AT*^2^ (inset of Fig. 1D). The quadratic temperature dependence of ρ, a characteristic of Fermi liquid, indicates the importance of the electron-electron correlation. The residual resistivity ratio (RRR ≡ ρ(300 K)/ρ_0_) of 260 for sample #1 and 630 for sample #2 is nearly 10 and 30 times larger than that reported in crystal with RRR = 22 (*24*), respectively. Hereafter, the samples with RRR = 22 (*24*), 260 (#1), and 630 (#2) will be referred to as samples in moderately clean, very-clean, and ultraclean regions, respectively. It should be noted that clear quantum oscillations are reported in the sample with RRR = 220 (*30*). These confirm the high quality of the present crystals. By comparing these crystals with different RRRs, we can obtain pivotal information about the quasiparticle excitations that are intimately related to the superconducting gap function.

The upper critical fields determined by the resistive transition of sample #1 for ***H***‖*a* and ***H***‖*c*, $H_{c2}^{a}$ , and $H_{c2}^{c}$, respectively, are displayed in Fig. 1E. At zero temperature, $H_{c2}^{a}(0)$ and $H_{c2}^{c}(0)$ are approximately 12 and 17 T, respectively. The upper critical fields of sample #2 is close to those of #1. As shown in the inset, $H_{c2}^{a}$ is very close to $H_{c2}^{c}$ in the vicinity of *T_c_*. Because the initial slope at *T_c_* is proportional to the orbital limiting field, ${-({\mathrm{dH}}_{c2}^{a,c}/\mathrm{dT})|}_{T_{c}}\propto 1/\xi_{b}\xi_{c,a}$, where ξ*_a_*, ξ*_b_*, and ξ*_c_* are the coherence length along the *a*, *b* and *c* axes, respectively, the present results indicate ξ*_a_* ≈ ξ*_c_*. This suggests that the average Fermi velocity along the *a* axis is close to that along the *c* axis.

The heat capacity *C* of sample #1 exhibits a very sharp transition at *T_c_* with no peak splitting (fig. S1). For comparison, we plot the reported data for a very clean crystal with *T_c_* = 2.1 K (*30*), whose RRR = 220 is close to sample #1, and for a crystal with *T_c_* = 1.7 K (*20*). The temperature dependence of *C*/*T* for sample #1 is very close to the data of the very clean crystal with a similar RRR that shows a negligibly small residual *C*/*T* in the zero temperature limit. These results further support the high quality of the present crystals. The electronic heat capacity coefficient in the normal state γ is 120 mJ/K^2^ mol. The Kadowaki-Woods ratio, *A*/γ^2^ ≈ 2 × 10^−5^ microhm·cm (mol K/mJ)^2^, is close to that of typical correlated systems (*33*).

**Fig. 2.**
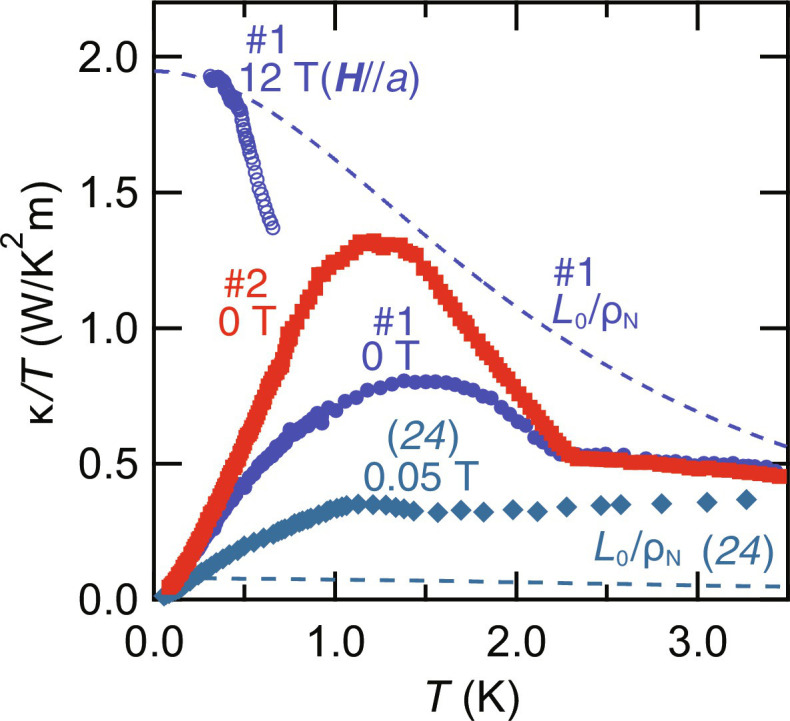
Temperature dependence of thermal conductivity of UTe_2_. In zero field, κ/*T* shows a kink at *T_c_* and then increases up to ∼1.5 and ∼1.2 K for samples #1 and #2, respectively. The blue dashed line represents the electronic contribution estimated by *L*_0_/ρ_N_ for sample #1. At low temperatures, *L*_0_/ρ_N_ is close to the normal state value of κ/*T* at 12 T for ***H***‖*a* (blue open circles), indicating that κ is dominated by the electronic contribution. For comparison, we plot κ/*T* at 0.05 T and *L*_0_/ρ_N_ for the moderately clean crystal with RRR = 22 (*24*).

Figure 2 shows the *T*-dependence of κ/*T* with applied thermal current ***Q***‖*a* for the very clean (#1) and ultraclean (#2) crystals, along with κ/*T* for the moderately clean crystal (*24*). In the normal state, both electron and phonon contribute to the thermal conductivity. To evaluate the electron contribution in the normal state at very low temperatures, we measure the normal state thermal conductivity κ_N_/*T* (open blue circles) by applying magnetic fields of μ_0_*H* = 12 T, which exceeds $H_{c2}^{a}$. The blue dashed line represents the electron contribution yielded from the Wiedemann-Franz (WF) law *L*_0_/ρ_N_. Here, *L*_0_ = 2.44 × 10^−8^ W·ohm·K^−2^ is the Lorenz number, and the normal state resistivity ρ_N_ below *T_c_* is calculated from the extrapolation above *T_c_* by assuming ρ_N_(*T*) = ρ_0_ + *AT*^2^. As shown in Fig. 2, κ_N_/*T* well coincides with *L*_0_/ρ_N_ below 0.5 K, indicating that the WF law holds. This demonstrates that, for the very clean and ultraclean crystals, the electron contribution is dominant in the normal state at very low temperatures. In zero field, κ/*T* exhibits a distinct kink upon entering the superconducting state, increases steeply, and reaches a maximum at ∼1.5 and ∼1.2 K for samples #1 and #2, respectively. As observed in several strongly correlated systems (*34*–*36*), the enhancement of κ/*T* below *T_c_* is attributed to the rapid enhancement of the quasiparticle mean free path, which is caused by the suppression of the electron-electron inelastic scattering rate due to the superconducting gap formation. The enhancement of κ/*T* of sample #2 is more substantial than sample #1 because the mean free path is larger in samples with larger RRR. We note that the enhancement of κ/*T* in the moderately clean crystal is much smaller than in these crystals. Moreover, κ_0N_/*T* ≡ κ_N_/*T* (*T* → 0) of sample #1 determined by the data taken at 12 T is 1.9 W/K^2^ m, one order of magnitude larger than 0.1 W/K^2^ m reported for the moderately clean crystal (*24*). The difference of κ_0N_/*T* also reflects the significantly enhanced mean free path in the very clean and ultraclean crystals.

**Fig. 3.**
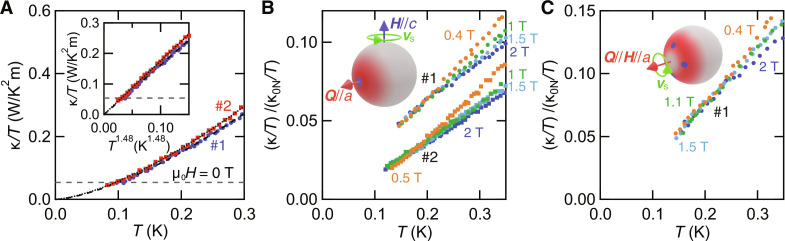
Temperature dependence of thermal conductivity of UTe_2_ at low temperatures. (**A**) Thermal conductivity divided by temperature, κ/*T*, in zero field for the very clean (#1) and ultraclean (#2) crystals. The data are extrapolated to *T* = 0 by κ/*T* = κ_0_/*T* + *AT*^α^ with κ_0_/*T* ≥ 0 (black dashed and dotted lines), yielding κ_0_/*T* ≈ 0 and α = 1.48 for both samples. The inset displays κ/*T* and the fit as a function of *T*^1.48^. The gray dashed horizontal lines represent the universal constant for line nodes (see Results section). (**B** and **C**) Temperature dependence of κ/*T* normalized by the normal state value in the zero temperature limit κ_N0_/*T* for magnetic fields ***H*** parallel to the crystal *c* axis (B) and *a* axis (C) with applied thermal current ***Q***‖*a*. The normal state value κ_0N_/*T* of the very clean crystal for ***H***‖*a* is determined by the data at 12 T (blue open circles in Fig. 2). κ_0N_/*T* for other configurations is approximated by *L*_0_/ρ_0_. As illustrated in the inset, κ/*T* for ***H***‖*c* (***H***‖*a*) selectively probes the quasiparticles in the red-shaded area and is sensitive to the nodes (blue circles) at (around) the *a* axis.

Figure 3A shows the *T*-dependence of κ/*T* at low temperatures for samples #1 and #2. The thermal conductivity in the superconducting state has quasiparticle and phonon contributions, κ = κ_qp_ + κ_ph_. In the boundary-limited scattering regime at low temperatures, κ_ph_/*T* is proportional to ℓ_ph_*T*^2^, where ℓ_ph_ is the phonon mean free path. While ℓ_ph_ is *T*-independent for diffuse scattering limit, resulting in κ_ph_/*T* ∝ *T*^2^, it is *T*^−1^-dependent for specular reflection, resulting in κ_ph_/*T* ∝ *T*. In real systems, κ_ph_/*T* ∝ *T^p^* with 1 ≤ *p* ≤ 2. Because κ_ph_/*T* becomes zero at *T* = 0, κ_0_/*T* ≡ κ/*T* (*T* → 0) contains only the quasiparticle contribution. It is apparent the linear extrapolation of κ/*T* to *T* = 0 yields a negative intercept. We find that κ/*T* for both samples is best fitted by κ/*T* ∝ *T*^α^ with α = 1.48 below 0.3 K (inset of Fig. 3A). These results indicate the absence of residual thermal conductivity κ_0_/*T* ≈ 0. For unconventional superconductors with line nodes in the energy gap, a finite residual term is expected because of the existence of a residual normal fluid (*37*). Therefore, the present results provide evidence for the absence of line nodes. This is further supported by the estimation of the residual term in a line-nodal superconductor. For line nodes, the universal expression of κ_0_/*T* for unitary scattering is given by $\frac{L_{0}}{\mu_{0}\lambda^{2}}\frac{\hbar }{\pi \Delta_{0}}$, where μ_0_ is the permeability of vacuum and λ is the penetration depth. Using 2Δ_0_ = 3.5*k*_B_*T_c_* and λ ≈ 1000 nm (*24*, *25*), we obtain κ_0_/*T* ≈ 0.054 W/K^2^ m (gray dashed line). Clearly, κ/*T* at the lowest temperature is less than the calculated value for the line nodes.

We next examine the presence/absence of point nodes. It is well-known that the heat transport at low magnetic fields in nodal superconductors is fundamentally different from that in full-gap superconductors. In magnetic fields, the energy of quasiparticles with momentum *ℏ****k*** in a circulating supercurrent flow ***v***_s_ shifts as *E*_***k***_ → *E*_***k***_ − *ℏ****k*** · ***v***_s_. As a result of this Doppler shift, the thermal conductivity in nodal superconductors is dominated by the contribution from delocalized quasiparticles, leading to an initial steep increase of $\kappa \left(H\right)/T\propto \sqrt{H}$ for line nodes and κ(*H*)/*T* ∝ ∣*H* log *H*∣ for point nodes (*38*). By contrast, in full-gap type II superconductors in the clean limit, all quasiparticles are bound to the vortex cores, and the magnetic field has a negligible effect on the heat transport (*39*–*41*) except in the vicinity of *H*_*c*2_, where the vortex cores overlap.

Thermal conductivity is a directional probe sensitive to the quasiparticles with momentum parallel to the thermal current (***k*** · ***Q*** ≠ 0) and perpendicular to the magnetic field (***k*** × ***H*** ≠ 0) because ***H*** ⊥ ***v***_s_. To investigate the quasiparticle excitations around the *a* axis, we measured κ along the *a* axis (***Q***‖*a*) for ***H***‖*c* and ***H***‖*a* (Fig. 3, B and C). As seen in the insets, while the former geometry sensitively probes the point node at the *a* axis, the latter geometry selectively probes quasiparticle excitations from point nodes that are off *a* axis but have momentum in the *a*-axis direction. For the very clean crystal (#1), κ/*T* collapses into a single curve and decreases almost linearly below ∼0.25 K for both ***H***‖*c* and ***H***‖*a*. For the ultraclean crystal (#2), κ/*T* normalized by the normal state value κ_0N_/*T* for ***H***‖*c* is nearly half of that for the very clean crystal below ∼0.3 K (Fig. 3B). Simple linear extrapolations to *T* = 0 give negative intercepts for all data, indicating a very small residual κ/*T* at *T* = 0. To obtain the zero temperature limit of κ/*T* quantitatively, the data are fitted using κ/*T* = κ_0_/*T* + *AT*^α^ with κ_0_/*T* ≥ 0 and 1 ≤ α ≤ 2. This yields the vanishingly small κ_0_/*T* at low fields for both samples (figs. S2 to S4A).

**Fig. 4.**
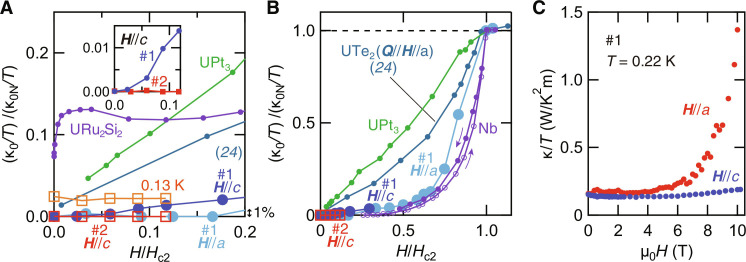
Field dependence of thermal conductivity of UTe_2_. (**A** and **B**) The residual thermal conductivity, κ_0_/*T*, normalized by the normal state value, κ_0N_/*T*, as a function of *H*/*H*_c2_. For both ***H***‖*c* and ***H***‖*a*, κ_0_/*T* is less than 1% of κ_0N_/*T* up to *H*/*H*_c2_ = 0.09, which provides evidence for the absence of any types of nodes at or around the *a* axis. Orange open squares depict (κ/*T*)/(κ_0N_/*T*) of sample #2 for ***H***‖*c* at *T* = 0.13 K (see also fig. S4B). The inset shows an enlarged view in the low field region for ***H***‖*c*. For comparison, we plot data for other uranium superconductors UPt_3_ (*42*) and URu_2_Si_2_ (*36*) (A) and typical s-wave superconductor Nb (*39*) and nodal superconductor UPt_3_ (*42*) (B). We also plot the data for the moderately clean crystal with RRR =22 for ***Q***‖***H***‖*a* (*24*). (**C**) Field dependence of κ/*T* at 0.22 K for ***H***‖*c* and ***H***‖*a* for sample #1.

Figure 4A shows κ_0_/*T* normalized by κ_0N_/*T* as a function of *H*/*H*_*c*2_. κ_0_/*T* of the very clean crystal (#1) is less than 1% of the normal-state value even at $H∼0.09\;H_{c2}^{c}$ for ***H***‖*c* and at $H∼0.2\;H_{c2}^{a}$ for ***H***‖*a*. Moreover, as shown in the inset, (κ_0_/*T*)/(κ_0N_/*T*) of the ultraclean crystal (#2) for ***H***‖*c* is significantly suppressed from that of the very clean crystal. For comparison, we plot data for two other U-based superconductors, UPt_3_ (*42*) and URu_2_Si_2_ (*36*), which are believed to have both point and line nodes. In notable contrast to the present results, κ_0_/*T* for UPt_3_ and URu_2_Si_2_ exhibits a steep increase at low fields. As shown in Fig. 4A (see also fig. S4B), even at 0.13 K, κ/*T* of the ultraclean crystal is only 2% of κ_0N_/*T* up to $H∼0.12\;H_{c2}^{c}$, still much smaller than (κ_0_/*T*)/(κ_0N_/*T*) for UPt_3_ and URu_2_Si_2_. These results indicate that UTe_2_ is essentially different from other U-based unconventional superconductors in that only a very few delocalized quasiparticles are excited, even well inside the vortex state. In Fig. 4B, we compare κ_0_/*T* of UTe_2_ with typical s-wave superconductor Nb (*39*) and nodal superconductor UPt_3_ (*42*) over a wide field range. For Nb, we show the data measured in both ascending and descending magnetic fields, although the difference is small. Here, we restrict the argument of *H*-dependence for ***H***‖*c* to the low field regime, where the normal-state magnetoresistance is small and κ_0N_/*T* can be approximated by *L*_0_/ρ_N_ (fig. S5). It is evident that *H*-dependence of UTe_2_ is very close to that of Nb. These results lead us to conclude that quasiparticle excitations with the velocity component along the *a* axis are negligibly small, ruling out the presence of point nodes at and around the *a* axis. This indicates that κ in zero field is dominated by the phonon contribution κ_ph_ at low temperatures, consistent with the power law behavior κ/*T* ∝ *T*^1.48^ (see inset of Fig. 3A).

The field dependence of κ/*T* at 0.22 K for ***H***‖*c* and ***H***‖*a* (Fig. 4C), which are both nearly constant at low fields, further supports the absence of point nodes around the *a* axis. It has been reported that the thermal conductivity of the *d*-wave superconductor CeCoIn_5_ shows little *H*-dependence at very low temperatures (*35*, *43*). One possible explanation is that the $\sqrt{H}$ dependence of the quasiparticle density of states is canceled out by the reduction of the quasiparticle mean free path, which is proportional to intervortex distance $\propto 1/\sqrt{H}$ (*44*, *45*). However, such a cancellation is not expected for the full gap and point-nodal superconductors. Another possibility of the multigap effect has also been pointed out (*43*). However, it is highly unlikely that the vanishingly small and nearly field independent κ/*T* in ultraclean UTe_2_ is caused by the multiband effect.

## DISCUSSION

Having established the absence of any type of nodes at and around the *a* axis, we discuss the superconducting order parameter belonging to the irreducible representation of the *D*_2*h*_ point group in UTe_2_. Our results rule out the *B*_3*u*_ state with point nodes along the *a* axis. NMR measurements reported a clear reduction of the Knight shift for ***H***||*b* and ***H***||*c* (*11*, *12*). These rule out the possibilities of the *B*_1*u*_ and *B*_2*u*_ states. It should be noted that, because the Knight shift measurements for ***H***||*a* contain large error bars, it is premature to conclude the *B*_3*u*_ state pointed out in (*12*). On the basis of these results, we conclude that the superconducting order parameter in UTe_2_ is represented by the *A*_*u*_ symmetry.

The recent penetration depth measurements have proposed a *B*_3*u*_ + *i*ϵ*A_u_* state with ϵ comparable to unity (*26*). This state has point nodes close to the *b* and *c* axes, which may not be sensitively detected by the *a*-axis thermal conductivity. However, such a state can be safely ruled out because the double superconducting transitions, required for such a multicomponent state (*46*), have never been observed in clean UTe_2_ crystals (*18*–*20*), even in the presence of uniaxial strain (*21*).

Here, we comment on the results of the specific heat. The quadratic temperature dependence of *C*/*T* has been reported in the very clean crystal with RRR = 220 (*30*), suggesting the presence of point nodes. However, we point out that the heat capacity data of UTe_2_ need to be scrutinized because they include contributions from both local and itinerant excitations and may include local excitations that are not directly related to the quasiparticles excited from the superconducting condensate. Recent muon spin rotation experiments have reported that uranium defects induce local magnetic clusters that give a finite magnetic contribution to *C*/*T* (*47*). Given this situation in UTe_2_, thermal conductivity, which is not influenced by such local excitations, is a more direct bulk probe for determining the superconducting gap structure of this material.

In addition to the spin-triplet full-gap superconductivity, an unexpected and unique feature of the superconducting state of UTe_2_ is that the quasiparticle excitations are extremely sensitive to disorder due to impurities/defects. In Fig. 4A and fig. S6, we compare (κ_0_/*T*)/(κ_0N_/*T*) for crystals with different RRR as a function of *H*/*H*_*c*2_ and RRR, respectively. The data of RRR = 22 are taken from (*24*). In contrast to the present results, it has been pointed out that the field dependence of κ/*T* of the moderately clean crystal bears a resemblance to that of superconductors with point nodes (*24*). Moreover, as shown in the inset of Fig. 4A, (κ_0_/*T*)/(κ_0N_/*T*) of the ultraclean crystal (#2) is one order of magnitude smaller than that of the very clean crystal (#1) at *H*/*H*_*c*2_ ∼ 0.09, indicating that the quasiparticle excitations are still strongly affected by the disorder even in the ultraclean region where RRR is well above 260. It is well-known that impurities have a considerable effect on quasiparticle excitations in unconventional superconductors, and this appears to be more pronounced in UTe_2_. The extreme sensitivity of quasiparticle excitations to the disorder should provide important clues to the mechanism of superconductivity in UTe_2_ that still remains elusive. A plausible origin of the disorder is uranium defects whose concentration is very sensitive to sample growth conditions (*31*). It has been suggested that the uranium defects locally disrupt long-range magnetic correlations (*47*–*49*) that may have a large impact on the quasiparticle excitations.

We emphasize that the fully gapped *A_u_* symmetry is the realization of spin-triplet superconductivity analogous to the *B*-phase of superfluid ^3^He. The full-gap spin-triplet superconductivity has also been suggested in UBe_13_ (*50*, *51*). However, in UBe_13_, the information of the Fermi surface is almost absent, and notable deviation from the Fermi liquid behavior is observed in the normal state (*51*). Therefore, it is challenging to investigate the topological properties of UBe_13_. In contrast, for UTe_2_ that exhibits the conventional Fermi liquid behavior in the normal state, the topological properties are characterized by the shape of the Fermi surface (*29*).

It should be noted that UTe_2_ likely has the three-dimensional (3D) Fermi surface. Recent measurements of angle-resolved photoemission spectroscopy reveal the presence of 3D electronic structure with closed Fermi surface along the *c* axis (*52*, *53*). The 3D structure is further supported by the almost isotropic normal state resistivity (*54*) and the nearly isotropic coherence lengths along the *a* and *c* axes (Fig. 1E). We note that, although several experiments have reported a larger anisotropy of ξ*_a_* and ξ*_c_* by a factor of 2 to 3 (*27*, *55*, *56*), this does not change the argument of the dimensionality of the Fermi surface. Although very recent quantum oscillation measurements reported the presence of 2D Fermi surfaces (*30*), this does not exclude the 3D Fermi surface. This is because the topology of the Fermi surfaces may change in strong magnetic fields due to a Lifshitz transition (*57*). Moreover, the electronic specific heat coefficient γ*_e_* ∼ 100 mJ/K^2^ mol obtained from the quantum oscillations is still smaller than γ*_e_*∼ 120 mJ/K^2^ mol from the specific heat, implying the existence of undiscovered Fermi surface. Given the 3D Fermi surface, the *A_u_* state is characterized by a nontrivial 3D winding number (*29*). In this case, UTe_2_ is a long-sought 3D strong topological superconductor with emergent Majorana surface states on any crystal plane. After completion of this work, we became aware of NMR experiments on the ultraclean crystal (*58*). The reduction of the *a*-axis Knight shift is consistent with the fully gapped *A*_*u*_ symmetry.

## MATERIALS AND METHODS

High-quality single crystals of UTe_2_ were grown by a flux method as described in (*31*). We used two crystals #1 and #2 from different batches. Sample #1 was cleaved into two pieces. The data for sample #1 are primarily from measurements on one piece. The other piece of sample #1 was used for resistivity experiments to obtain the upper critical fields and magnetoresistance. The size of the former piece is 2511 μm (length) by 147.5 μm (width) by 77.5 μm (thickness). The size of the latter piece is 2230 μm (length) by 204.5 μm (width) by 75.3 μm (thickness). The size of sample #2 is 2467 μm (length) by 178.8 μm (width) by 104 μm (thickness). The resistivity was measured by a standard four-probe method by applying current along the crystal *a* axis. Four gold wires were attached by spot welding. The specific heat was measured by a long–relaxation time method as described in (*59*, *60*).

Thermal conductivity κ was measured by the standard steady-state method by applying temperature gradient ***Q*** along the crystal *a* axis. To obtain good contacts with low contact resistance of ∼0.5 ohms, four gold wires were attached by spot welding. The gold wires serve heat links to a 10-kilohm chip resistor as a heater, two field calibrated thermometers, and a silver plate. The silver plate was fixed with silver paste to a copper block as a heat bath.
