# Supplementary material for: Fully gapped pairing state in spin-triplet superconductor UTe2
Source: Sci Adv. 2024 Feb 7;10(6):eadk3772. doi: 10.1126/sciadv.adk3772 (PMC10849587; doi:10.1126/sciadv.adk3772)
Supplement: Supplementary file 1 — Figs. S1 to S6 [file sciadv.adk3772_sm.pdf]

Science Advances
AAAS

Supplementary Materials for

Fully gapped pairing state in spin-triplet superconductor UTe_2

Shota Suetsugu *et al.*

Corresponding author: Shota Suetsugu, suetsugu.shota.4n@kyoto-u.ac.jp;
Yuji Matsuda, matsuda@scphys.kyoto-u.ac.jp

Sci. Adv. **10**, eadk3772 (2024)
DOI: 10.1126/sciadv.adk3772

This PDF file includes:

Figs. S1 to S6

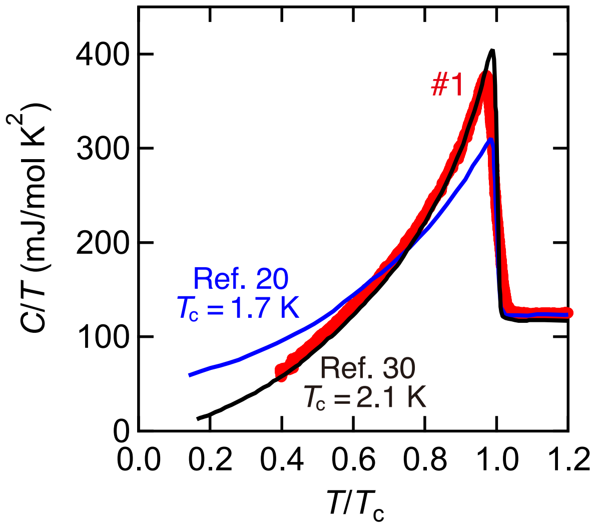

Fig. S1: Specific heat divided by temperature for the very clean crystal (#1) as a function of T/T_c. For comparison, we plot the data for a very clean crystal with $T_c = 2.1\,\mathrm{K}$ [30] and a crystal with $T_c = 1.7\,\mathrm{K}$ [20].

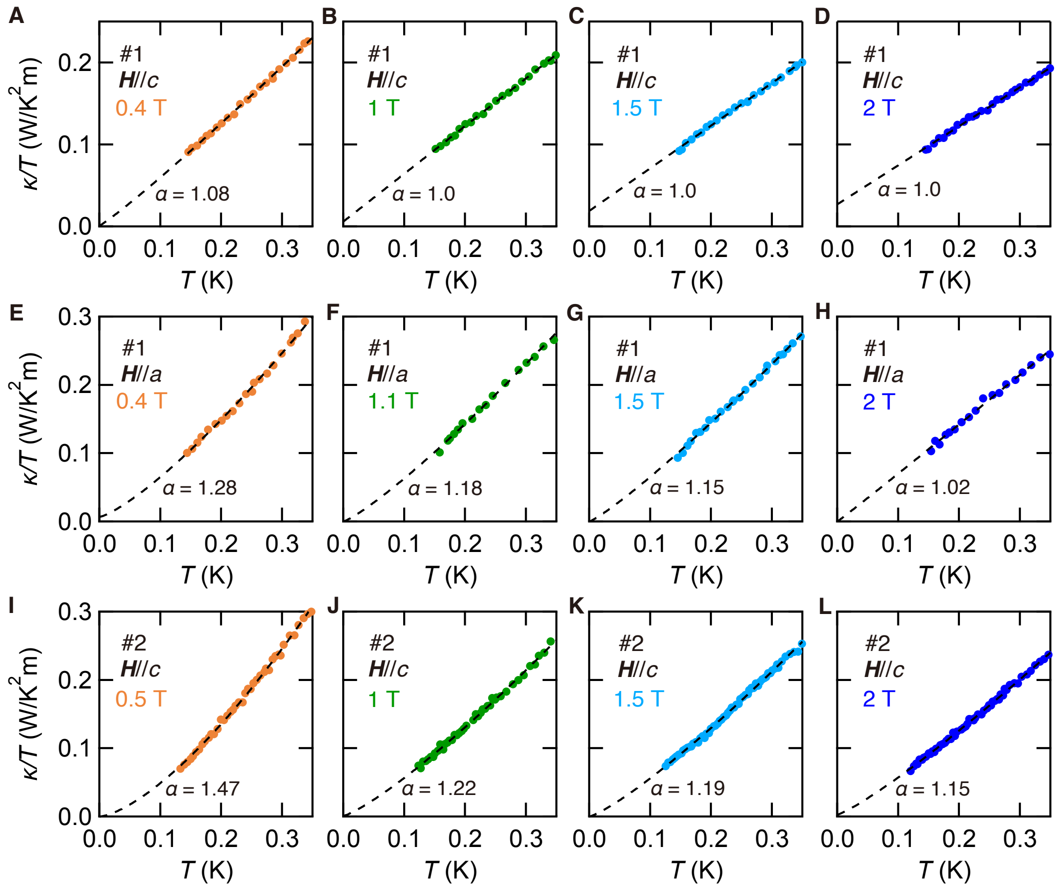

Fig. S2: Temperature dependence of thermal conductivity at low fields. (**A** to **D**) Thermal conductivity divided by temperature, κ/T, of sample #1 for $\boldsymbol{H}||c$ at 0.4, 1, 1.5, and 2 T. (**E** to **H**) κ/T of sample #1 for $\boldsymbol{H}||c$ at 0.4, 1.1, 1.5, and 2 T. (**I** to **L**) κ/T of sample #2 for $\boldsymbol{H}||c$ at 0.5, 1, 1.5, and 2 T. These data are extrapolated to $T = 0$ by $\kappa/T = \kappa_0/T + AT^{\alpha}$ (dashed lines) with α shown in each panel.

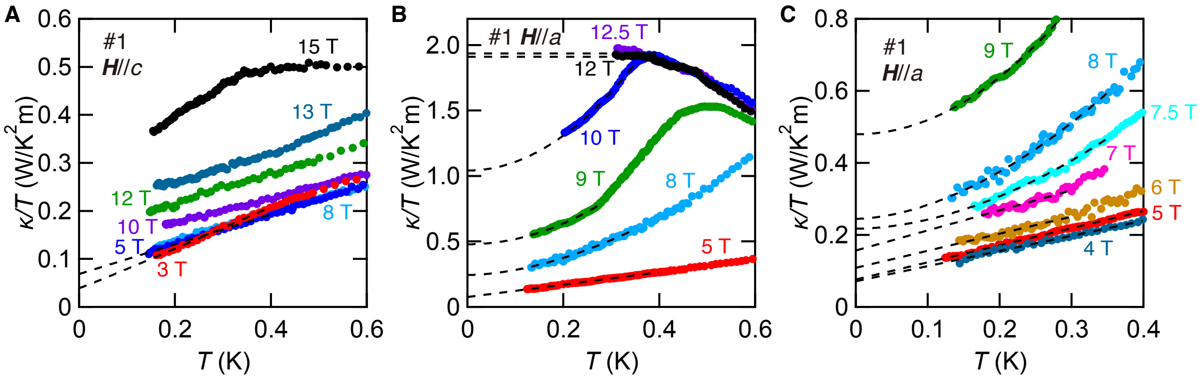

Fig. S3: Temperature dependence of thermal conductivity for sample #1 above 2 T. (**A** to **C**) Temperature dependence of κ/T for $\boldsymbol{H}||c$ (**A**) and $\boldsymbol{H}||a$ (**B** and **C**). These data are fitted by $\kappa/T = \kappa_0/T + AT^{\alpha}$ (dashed lines).

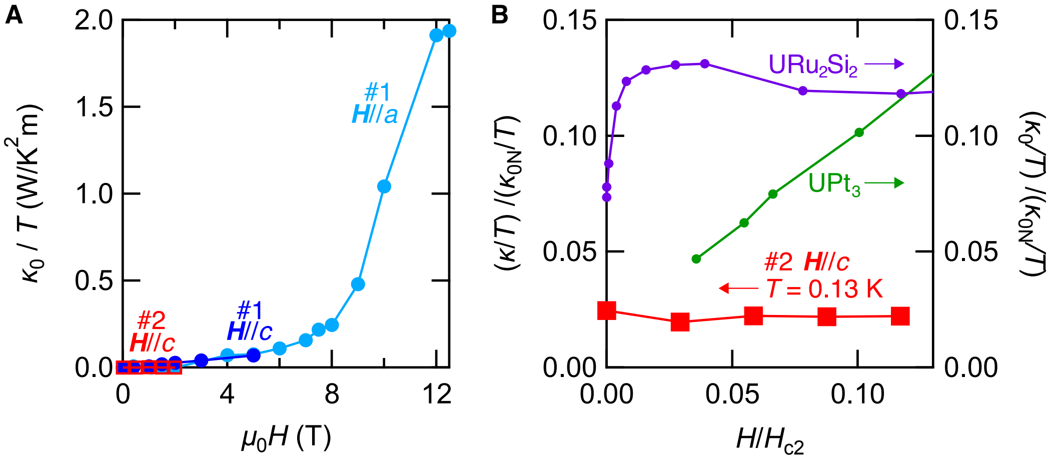

Fig. S4: Field dependence of thermal conductivity. (**A**) Field dependence of κ_0/T without normalization. (**B**) κ/T normalized by $\kappa_{0\mathrm{N}}/T$ at 0.13 K for sample #2 as a function of H/H_{c2}. For comparison, we plot κ_0/T normalized by $\kappa_{0\mathrm{N}}/T$ for UPt_3 [42] and URu_2Si_2 [36].

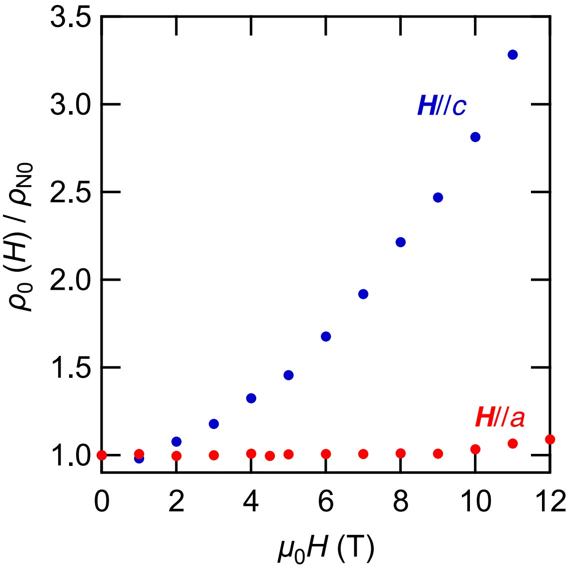

Fig. S5: Field dependence of residual resistivity $\rho_0(H)$ normalized by $\rho_{N0} \equiv \rho_0(H = 0)$ for sample #1. $\rho_0(H)$ is obtained by a fit to $\rho(H,T) = \rho_0(H) + A(H)T^2$. While $\rho_0(H)$ is nearly H-independent for $\boldsymbol{H}||a$, $\rho_0(H) \propto H^2$ is observed for $\boldsymbol{H}||c$. Because of small magnetoresistance $\rho_0(\boldsymbol{H}||c)/\rho_{N0} < 1.5$ for $H \leq 5\,\mathrm{T}$, κ_{0N}/T can be approximated by L_0/ρ_{N0}.

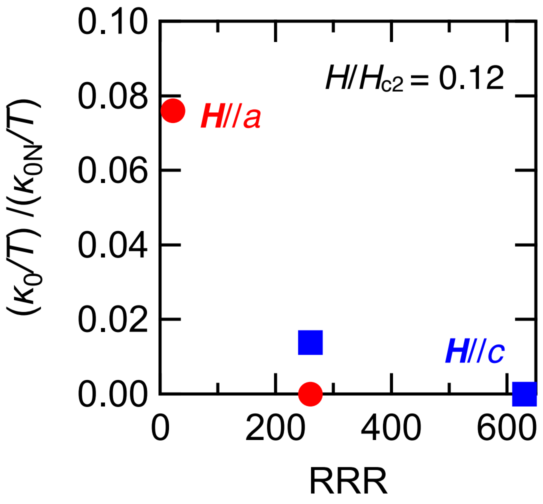

Fig. S6: κ_0/T normalized by κ_{0N}/T at $H/H_{c2} = 0.12$ for $\boldsymbol{H}||a$ (red circles) and $\boldsymbol{H}||c$ (blue squares) as a function of RRR. The value of RRR = 22 was linearly interpolated from data in the literature [24].
